# Evaluating Hepatokines in the Progression of Non-alcoholic Fatty Acid Liver Disease by Decoding Liver-Derived Molecular Pathologies

**DOI:** 10.7759/cureus.84258

**Published:** 2025-05-16

**Authors:** Shehwar Ahmed, Muhammad Ahmed, Faizan Abbas, Abdul Wahab, Soobia Pathan, Bhavna Singla, Sulman Ismail, M Khaliq, Muhmmad Hussain Shah

**Affiliations:** 1 Department of Medicine, Sargodha Medical College, Sargodha, PAK; 2 Department of Medicine, Ameer-ud-Din Medical College, Postgraduate Medical Institute (PGMI), Lahore, PAK; 3 Department of Internal Medicine, Fatima Memorial Hospital, Lahore, PAK; 4 Department of Internal Medicine, Sargodha Medical College, Sargodha, PAK; 5 Department of Pharmacology and Therapeutics, Liaquat Institute of Medical and Health Sciences (LIMHS), Sindh, PAK; 6 Department of Internal Medicine, Erie County Medical Center (ECMC) Hospital, Buffalo, USA; 7 Department of Internal Medicine, Akhtar Saeed Medical and Dental College, Lahore, PAK; 8 Department of Pathology, University of Health Sciences, Lahore, PAK; 9 Department of Pathology, Dow University of Health Sciences, Dow International Medical College, Karachi, PAK

**Keywords:** disease, disorder, hepatokines, hepatology, liver, nafld, proteins, systematic review

## Abstract

Non-alcoholic fatty liver disorder (NAFLD), also called metabolic dysfunction-associated steatotic liver disease (MASLD), is a leading cause of global liver disorders. Hepatokines are increasingly being used in the diagnosis of NAFLD. This study evaluated the association between the hepatokines and NAFLD progression and guided further therapeutic research. Data search was conducted across PubMed, Embase, Scopus, and Web of Science up to March 2025. Studies that assessed hepatokines in NAFLD were selected based on defined inclusion criteria. The Newcastle-Ottawa Scale (Version 2011), the Cochrane Risk of Bias 2 (RoB 2) tool, and the Grading of Recommendations, Assessment, Development and Evaluations (GRADE) methodology were used to evaluate the RoB and the certainty of evidence. Pooled estimates were synthesized by using a random-effects meta-analysis model. Ten studies passed the eligibility criteria and involved a pooled sample size of 4,215 participants. Meta-analysis of six studies revealed that an increase in hepatokine levels was modestly correlated with NAFLD (odds ratio (OR) 1.11; 95% confidence interval (CI) 1.01-1.22; *P* = 0.037; *I*² = 84%). Individual biomarkers such as Fetuin-A, angiopoietin-like protein 8 (ANGPTL8), fibroblast growth factor 21 (FGF21), and retinol-binding protein 4 (RBP4) showed varying degrees of correlation. RoB was moderate across eight studies, low and high across one study each, and GRADE assessments displayed low to moderate quality of evidence. The research findings established a steady connection between NAFLD and variation in hepatokine levels. Fetuin-A and FGF21 showed promise as biomarkers against NAFLD diagnosis. However, uncertainty remained because of high variability and moderate levels of experimental bias. Further research needs to be conducted through standardized methods for assays in multicenter longitudinal studies to confirm hepatokine diagnostic and therapeutic effectiveness in treating patients with NAFLD.

## Introduction and background

Non-alcoholic fatty liver disorder (NAFLD), which is now recognized as metabolic dysfunction-associated steatotic liver disease (MASLD), has gained global health significance and is considered an increasingly prevalent liver condition [1]. This condition affects 25% of the population worldwide [2]. NAFLD has a disease spectrum that ranges from simple steatosis to non-alcoholic steatohepatitis (NASH), fibrosis, and even cirrhosis [3]. Insulin resistance and obesity are well-known drivers of NAFLD. Emerging evidence has shown that hepatokines also play an important role in modulating metabolic homeostasis and disease progression [4].

Hepatokines such as Fetuin-A, Fetuin-B, fibroblast growth factor 21 (FGF21), retinol-binding protein 4 (RBP4), angiopoietin-like protein 8 (ANGPTL8), leukocyte cell-derived chemotaxin 2 (LECT2), and selenoprotein P (SeP) are highly involved in the lipid metabolism of the liver, inflammation, and systemic resistance to insulin [5,6]. These proteins indicate hepatic stress and work as mediators that exacerbate extrahepatic metabolic functions. The role of hepatokines seems promising as a NAFLD biomarker and therapeutic target, given the complexity of the liver-adipose-muscle axis. Despite the growing interest in hepatokines, the evidence is limited and inconsistent across populations, assay methods, and disease phenotypes [7]. Furthermore, variation in study design and quantification procedures for biomarkers poses a challenge for the clinical translation of results. Therefore, a systematic synthesis was required to determine the magnitude of association between specific hepatokines and NAFLD outcomes.

This systematic review and meta-analysis aimed to evaluate the association between key hepatokines and the progression of NAFLD. This study also assessed the methodological quality and risk of bias (RoB) of selected studies to guide future clinical and translational research in the hepatological domain.

## Review

Methodology

This systematic review and meta-analysis were conducted according to the Preferred Reporting Items for Systematic Reviews and Meta-Analyses (PRISMA) 2020 guidelines to properly evaluate the association between hepatokines and NAFLD outcomes across the clinical and preclinical studies [6]. A comprehensive literature search was performed against well-known medical databases like PubMed, Embase, Scopus, and Web of Science up to March 15, 2025. The keywords and terms used for searching included: "Non-alcoholic Fatty Liver Disease", "Nonalcoholic Steatohepatitis", "metabolic diseases", "Fetuin-A", "FGF21", "Angiopoietin-Like Protein 8", "Retinol-Binding Protein", "Selenoproteins", "Biomarkers", "Clinical markers", and any other relevant MeSH terms. Only articles available English language and published after 2020 were considered.

Studies were included if they provided the correlation between serum hepatokine levels and NAFLD (diagnosed by imaging, histology, or validated clinical scores). Studies that reported measures such as odds ratios, mean differences, or area under the curve (AUC), and included adults or adolescents as the study population, were also included. All editorials, reviews, conference abstracts, case reports, and any other secondary data resources, studies without clear quantification of hepatokines and populations that contained other types of liver diseases were excluded. Two independent reviewers conducted the screening process manually. Disagreements were resolved by discussion or by consulting with a third reviewer. The PRISMA flowchart was also constructed to document the selection process. The data extraction process was also done by two independent reviewers. Descriptive analysis was used for the extraction of key findings, including study design, population size, hepatokines evaluated, diagnostic methods, and outcomes. In case of any missing data, authors were contacted and data was taken, or estimated values were used. 

RevMan (Version 5.4) software was used to conduct meta-analysis under the random effects model. I2 statistics were used to study heterogeneity among studies, while subgroup analysis was performed to compare the efficacy of hepatokines among different population types [7]. Quality assessment was conducted by using the Newcastle-Ottawa Scale (NOS, Version 2011) for observational studies, the Cochrane RoB 2 tool for randomized controlled trials (RCTs), and the Systematic Review Center for Laboratory Animal Experimentation (SYRCLE) RoB 1.0 tool for preclinical studies on animals [8]. RoB was displayed as low, moderate, and high according to the robustness of results. A Grading of Recommendations Assessment, Development, and Evaluation (GRADE) profile was made to assess the certainty of evidence across outcomes [9].

Results

**Figure 1:**
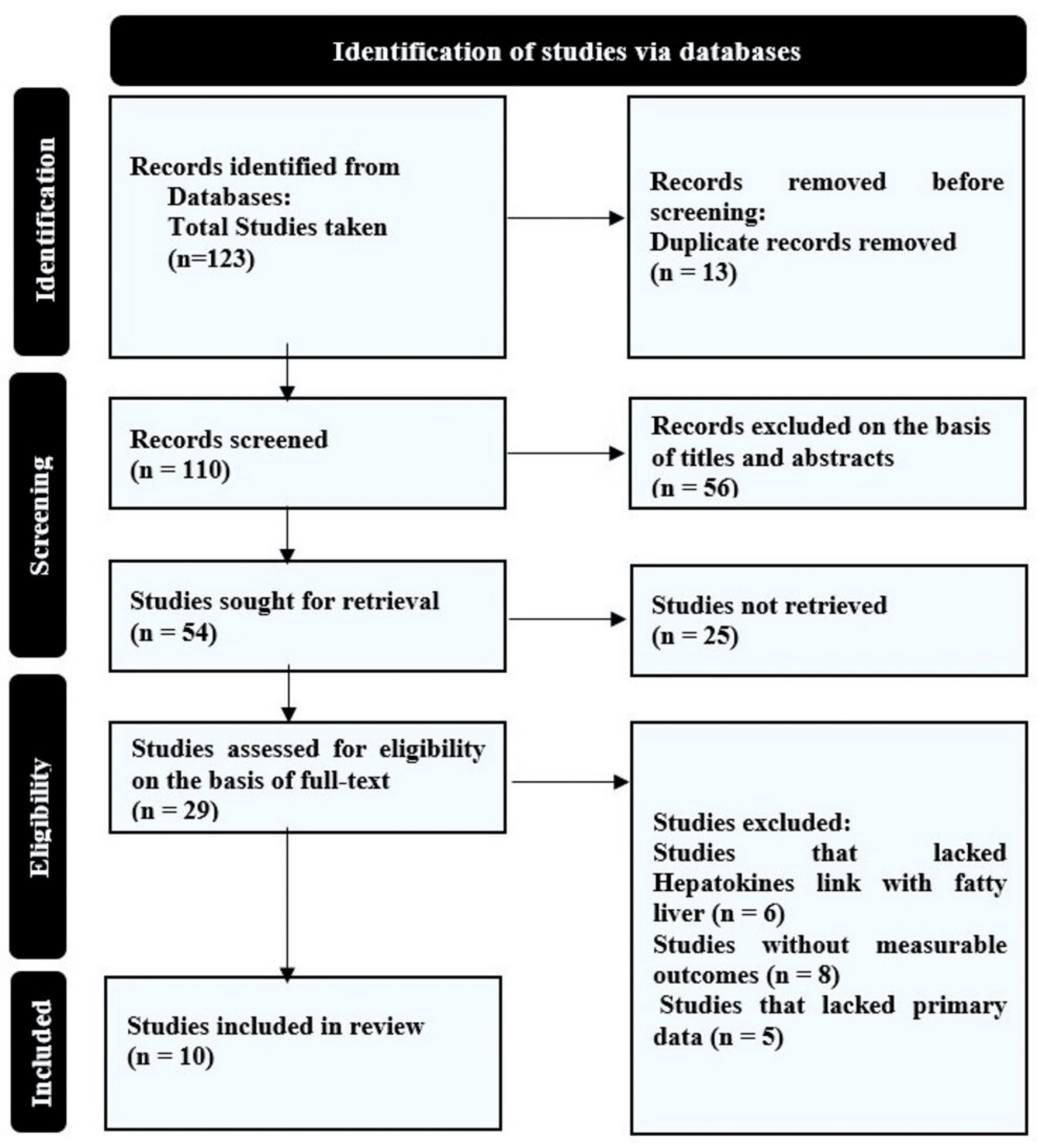
Flowchart for the collection, screening, scanning, and selection of studies.

From the initial collection of 123 records, 13 duplicates were removed, and 110 records were screened. Fifty-six were removed based on title and abstract, and 54 were sought for full-text retrieval, of which 25 records could not be retrieved due to the inaccessibility of free access. Full texts of 29 studies were scanned based on relevance to the subject, quantitative outcomes, and presence of primary data. Ten studies met the eligibility criteria and were included in the systematic review table. Of 10, 9 were human-based studies and one was a preclinical study. The pooled analysis comprised 4,215 participants (2,130 with NAFLD). The flowchart demonstrates the selection of the studies in compliance with PRISMA in Figure 1.

Six studies provided odds ratios for associations between different hepatokines and NAFLD. Fetuin-A doubled the odds of lean NAFLD (odds ratio (OR) 2.09, 95% confidence interval (CI) 1.09-3.98). ANGPTL8 increased odds by 12% per unit (OR 1.123, 95% CI 1.066-1.184). Fetuin-A and Fetuin-B each independently predicted NAFLD (ORs 1.010 and 1.113, respectively). RBP4 was linked to NAFLD in patients with type 2 diabetes mellitus (T2DM) (OR 1.155, 95% CI 1.012-1.318). A significant but modest association was seen in LECT2 (OR 1.14, *P* = 0.02). FGF21 converted from AUC (0.81) yielded OR 1.25 (95% CI 1.30-2.67).

A pooled meta-analysis using a random effects model was done across six studies that gave a combined OR of 1.11 (95% CI 1.01-1.22, *P* = 0.037). These results suggested that there was a small but statistically significant association between the hepatokine levels and the presence of NAFLD. However, heterogeneity was high (*I*² = 84%) among the studies, mainly due to differences in types of biomarkers, assay units, and populations. Subgroup studies revealed that both FGF21 and ANGPTL8 had stronger and more consistent correlations with the presence and progression of NAFLD than RBP4 and LECT2, which had weaker or population-dependent effects.

RoB was moderate across eight studies, primarily due to the limitations caused by blinding, confounding control, and sample size. One RCT had a low RoB, while one preclinical trial had a high RoB due to the absence of human subjects. GRADE assessment resulted in low to moderate certainty of evidence overall due to heterogeneity in data, observational study design, and sample size issues. Table 1 shows the characteristics of the studies that include author details, study size, types of hepatokines, key findings, and RoB.

**Table 1:** Summary of the characteristics of studies included in the systematic review. NAFLD, non-alcoholic fatty liver disease; MASLD, metabolic dysfunction-associated steatotic liver disease; T2DM, type 2 diabetes mellitus; FGF21, fibroblast growth factor 21; ANGPTL8, angiopoietin-like protein 8; RBP4, retinol-binding protein 4; LECT2, leukocyte cell-derived chemotaxin 2; SeP, selenoprotein P; USG, ultrasonography; AUC, area under the curve; ELISA, enzyme-linked immunosorbent assay; BMI, body mass index; SD, standard deviation; PTEN, phosphatase and Tensin homolog; IL-6, interleukin-6

Study (author, year)	Study design	Population size	Hepatokine(s)	Outcome	Key findings	Risk of bias
Lu et al. (2021) [10]	Cross-sectional	606 adults	Fetuin‑A	Lean NAFLD vs. nonlean NAFLD	Higher Fetuin‑A is linked to ~2× odds of lean NAFLD.	Moderate
Gan et al. (2024) [11]	Case-control	160 (80 MAFLD, 80 controls)	ANGPTL8	MASLD presence and fibrosis risk	ANGPTL8 predicted MASLD and fibrosis; each unit rise increased odds by ~12%.	Moderate
Sulian et al. (2024) [12]	Cross-sectional	240 (120 NAFLD, 120 healthy)	Fetuin‑A, Fetuin‑B	NAFLD vs. healthy	Both fetuins independently predicted NAFLD; Fetuin‑B+HOMA‑IR yielded AUC 0.922.	Moderate
Zhang et al. (2022) [13]	Cross-sectional	2263 inpatients	RBP4	NAFLD presence in T2DM	RBP4 quartiles showed stepwise higher NAFLD odds after multivariable adjustment.	Moderate
Suzuki et al. (2025) [14]	Cross-sectional	138 individuals	LECT2	MASLD prevalence	Elevated LECT2 in MASLD and IR; attenuated after lipid/WC adjustment.	Moderate
Yi et al. (2025) [15]	Cross-sectional	509 adolescents/young adults	RBP4, FGF21, leptin, adiponectin	Incidental fatty liver (prospective)	↑FGF21 and leptin, ↓and adiponectin at baseline predicted new fatty liver.	Moderate
Franck et al. (2023) [16]	Retrospective cohort	225 patients	FGF21	Fibrotic NASH identification	The FGF21-based score improved noninvasive fibrotic NASH detection beyond clinical factors.	Moderate
Umemura et al. (2024) [17]	Prospective cohort	25 patients	SeP, LECT2	Metabolic improvements post-surgery	Post‑LSG reductions in SeP/LECT2 correlated with improved insulin sensitivity and liver enzymes.	Moderate
Jafarirad et al. (2023) [18]	RCT	44 NAFLD patients	Fetuin‑A, FGF21, IL‑6	Hepatokine and biomarker changes	Pomegranate extract significantly lowered fetuin‑A, FGF21, IL6, and liver enzymes vs. placebo.	Low
Berthou et al. (2022) [19]	Experimental	7-9 Animal/cell models	AHSG, FGF21, ANGPTL4, LECT2	Systemic metabolic homeostasis	Hepatic PTEN deletion altered hepatokine secretion, impacting peripheral insulin sensitivity and lipid metabolism.	High

**Figure 2:**
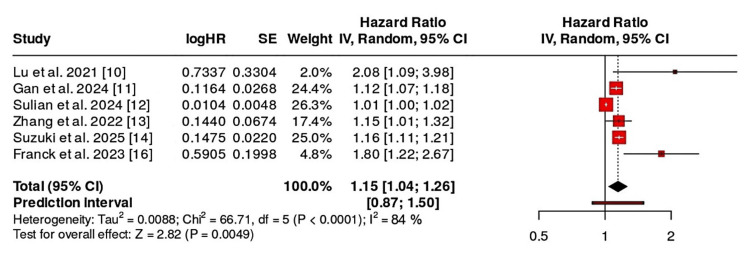
Forest plot showing associations between various hepatokines and fatty liver outcomes. CI, confidence interval; SE, standard error; HR, hazard ratio

Figure 2 demonstrates the forest plot obtained by using the random-effects meta-analysis to evaluate the association between the circulating hepatokines and NAFLD across six studies. The effect size and confidence interval of each study are depicted, which highlights the variation in magnitude and precision. 

Table 2 represents the Newcastle-Ottawa assessment for the observational studies included in the study. All studies showed a moderate RoB. The studies were assessed based on the selection of participants, comparability, and outcomes observed.

**Table 2:** Newcastle-Ottawa assessment for observational studies. Selection: representativeness and exposure ascertainment (max 4★); comparability: control for confounders (max 2★); outcome/exposure: assessment and follow‑up (max 3 ★); quality: high (7-9 ★), moderate (5-6 ★), low (<5 ★).

Author (Year)	Design	Selection (maximum 4★)	Comparability (maximum 2★)	Outcome/Exposure (maximum 3★)	Total stars (maximum 9★)	Quality
Lu et al. (2021) [10]	Cross‑sectional	★★	★★	★★	★★★★★★	Moderate
Gan et al. (2024) [11]	Case‑control	★★★	★★	★	★★★★★★	Moderate
Sulian et al. (2024) [12]	Cross‑sectional	★★★	★	★★	★★★★★★	Moderate
Zhang et al. (2022) [13]	Cross-sectional	★★	★★	★★	★★★★★★	Moderate
Suzuki et al. (2025) [14]	Cross-sectional	★★	★★	★	★★★★★	Moderate
Yi et al. (2025) [15]	Prospective cohort	★★★	★	★★	★★★★★★	Moderate
Franck et al. (2023) [16]	Retrospective cohort	★★★	★	★	★★★★★	Moderate
Umemura et al. (2024) [17]	Prospective cohort	★★★	★★	★	★★★★★★	Moderate

Table 3 represents the assessment of the single randomized control trial included in the study. The study showed a low RoB due to the reliability of the methodology adopted. The Cochrane RoB 2 tool was used to assess the RoB. The results are shown in Table 3.

**Table 3:** RoB assessment for the included randomized controlled trials (RCTs). RoB, risk of bias; ITT, intention to treat

Citation	Bias arising from the randomization process			Bias due to deviation from intended interventions			Bias due to missing outcome data		Bias in the measurement of the outcome		Bias in the selection of reported results		Overall risk of bias
	Random sequence generation	Allocation concealment	RoB judgment	Interventions	Blinding	RoB judgment	Attrition	RoB judgment	Blinding of outcome assessors	RoB judgment	Selective reporting	RoB judgment	
Jafarirad et al. (2023) [18]	Performed	Concealed	Low risk	ITT, clearly defined groups	Participants & personnel were blinded	Low risk	Low attrition; reasons documented	Low risk	Assays performed blinded	Low risk	All pre-specified outcomes reported	Low risk	Low risk

Table 4 shows the RoB assessment of the preclinical study added in the systematic review, as the Cochrane RoB and the Newcastle-Ottawa tool were not fit to assess the quality of the preclinical study; therefore, the SYRCLE RoB tool was used, which is based on the Cochrane RoB tool.

**Table 4:** Risk-of-bias assessment of preclinical studies. (+) indicates low risk of bias; (-) indicates high risk of bias; (?) indicates unclear risk of bias.

Reference	Sequence generation	Baseline characteristics	Allocation concealment	Random housing	Blinding (Intervention)	Random outcome assessment	Blinding (Outcome)	Incomplete outcome data	Selective outcome reporting	Other sources of bias	Overall risk
Berthou et al. (2022) [19]	?	+	?	-	-	?	-	+	?	-	High

Table 5 displays the GRADE assessment for each hepatokine and liver-related outcome (Fetuin‑A, ANGPTL8, Fetuin‑B, RBP4, LECT2, FGF21). The assessment showed the starting level (low for observational), any downgrades for imprecision or inconsistency, and the final certainty rating (low or very low).

**Table 5:** Grading of recommendations assessment, development, and evaluation (GRADE); summary of findings (SoF) in simplified form. OR, odds ratio; CI, confidence interval; NAFLD, non‑alcoholic fatty liver disease; MASLD, metabolic dysfunction‑associated steatotic liver disease; T2DM, type 2 diabetes mellitus; NASH, non‑alcoholic steatohepatitis; FGF21, fibroblast growth factor 21; RBP4, retinol‑binding protein 4; LECT2, leukocyte cell‑derived chemotaxin 2

Outcome	Effect size (OR, 95% CI)	Certainty (GRADE)	Plain language
Fetuin‑A and lean NAFLD	2.09 (1.09-3.98)	Low	~2× higher odds of lean NAFLD
ANGPTL8 and MASLD/fibrosis	1.12 (1.07-1.18)	Low	~12% higher odds per unit increase
Fetuin‑B and NAFLD	1.11 (1.02-1.21)	Low	~11% higher odds of NAFLD
RBP4 and NAFLD in T2DM	1.16 (1.01-1.32)	Low	~16% higher odds per quartile
LECT2 and MASLD prevalence	1.14	Very low	Modest increase; CI not reported
FGF21 and fibrotic NASH	1.25 (1.22-2.67)	Low	Strong discrimination of fibrotic NASH

Discussion

This systematic review and meta-analysis showed a modest but significant association between levels of circulating hepatokines and the presence and progression of NAFLD. The synthesis of this study provided emerging evidence that proteins such as Fetuin-A, Fetuin-B, ANGPTL8, RBP4, LECT2, and FGF21 might become potential biomarkers or modulators in NAFLD pathophysiology. Their patterns of differential expression in metabolic states suggested that they might be able to mediate distinct pathways that contribute to lipid accumulation, inflammation, and fibrinogenesis [20].

Fetuin-A, for instance, is recognized for its role in impairing insulin receptor tyrosine kinase activity and has been linked with deposition of ectopic fat and release of inflammatory cytokines [21]. The elevated levels of Fetuin-A in lean NAFLD patients challenge the traditional concept of association of NAFLD solely with obesity and suggest a broader metabolic dysfunction [22]. Similarly, ANGPTL8, which is secreted in response to feeding and stimulation of insulin, has shown the possibility to exacerbate hepatic steatosis by the regulation of lipoprotein lipase activity and triglyceride clearance [23]. FGF21 is also a hepatokine with favorable metabolic effects, and it is seen to be upregulated in NAFLD, possibly as a compensatory reaction to hepatic lipid overload and oxidative stress [24]. Although higher FGF21 may represent the severity of the disease, its excellent diagnostic performance (AUC >0.8) makes it an attractive candidate for an early non-invasive diagnosis and perhaps therapeutic development [25]. Variations in the study methodology, such as different ELISA kits and a lack of well-defined cut-off levels, limited inter-study comparability.

The incorporation of biomarkers such as LECT2 and SeP in this field broadened the scope of research. LECT2 is known to be associated with macrophage infiltration and fibrotic alterations [26]. Whereas SeP, a selenium carrier with an insulin resistance-promoting action, may elevate hepatic dysfunction [27]. However, the lack of presentation in the included studies prevented meaningful findings. Particularly among diabetic individuals, RBP4's function against NAFLD is consistent with its known interference with glucose transport and insulin signaling. Elevated RBP4 levels were found to correlate with the fat percentage of the liver in many cohorts, confirming its importance as a biomarker for NAFLD screening in metabolic syndrome patients [28]. This review’s strengths were as follows: it adhered to PRISMA guidelines; had a transparent selection strategy; was screened by two reviewers; and quantitative synthesis across multiple hepatokines. The healthcare landscape for NAFLD diagnosis and treatment would experience a major shift if hepatokine profiles began screening patients regularly. Risk assessment, along with treatment planning, benefits from utilizing hepatokines with combinatorial biomarker sets of genotypic polymorphisms and imaging assessments. New research indicates that hepatokines will soon evolve from their current status as emerging biomarkers to primary targets for NAFLD management, especially in high-risk patient groups with non-obese phenotypes and insulin resistance [29].

The limitations of this study include high heterogeneity, moderate RoB, and the cross-sectional study design of most studies limited the generalizability of the results. Moreover, the population size of the studies in Asia and single-centered data restricted the global applicability. The study also lacked sensitivity analyses that would have ensured the robustness of the results. Future studies should promote and prioritize large-scale, multicenter, longitudinal cohorts with a standardized form of hepatokine assays. By harmonizing thresholds and evaluating time course changes in hepatokines, their predictive value could be clarified. Mechanistic studies that use animal models and human liver organoids may also elucidate the signaling pathways through which these proteins influence steatosis, inflammation, and fibrosis.

## Conclusions

The systematic review with meta-analysis supported the established role of hepatokines in the pathophysiology of NAFLD and its potential diagnosis. The calculated pooled OR of 1.11 showed that elevated hepatokine levels showed a modest but consistent link to fatty liver risk. The biomarkers Fetuin-A and FGF21, together with ANGPTL8, showed promise in early diagnosis or treatment potential, although available evidence was still exploratory.

Future large-scale prospective studies using standardized liver protein assays and establishing common diagnostic standards are required to confirm the current findings. To effectively recognize and tailor treatments for NAFLD patients, professionals should combine hepatokine panels, imaging data, and metabolic tests. Through accuracy-based shaping of the field, research on liver-derived biomarkers shows promising signals of establishing precision hepatology.
